# In Vivo Total Ankle Arthroplasty Kinematic Evaluation: A Prospective Radiostereometric Analysis

**DOI:** 10.3390/biomedicines12040705

**Published:** 2024-03-22

**Authors:** Silvio Caravelli, Laura Bragonzoni, Raffaele Zinno, Emanuele Vocale, Erika Pinelli, Giuseppe Barone, Giulio Vara, Stefano Di Paolo, Stefano Zaffagnini, Massimiliano Mosca

**Affiliations:** 1 U.O. Ortopedia Bentivoglio, IRCCS Istituto Ortopedico Rizzoli, 40100 Bologna, BO, Italy; emanuelevocale@gmail.com (E.V.); massimiliano.mosca@ior.it (M.M.); 2 Dipartimento di Scienze della Qualità della Vita, University of Bologna, 40139 Bologna, BO, Italy; laura.bragonzoni4@unibo.it (L.B.); raffaele.zinno2@unibo.it (R.Z.); erika.pinelli2@unibo.it (E.P.); giuseppe.barone8@unibo.it (G.B.); stefano.dipaolo4@unibo.it (S.D.P.); 3 U.O. Radiodiagnostica, Ospedale Umberto I, 48022 Lugo, BO, Italy; giulio.vara@gmail.com; 4 II Clinic of Orthopaedics and Traumatology, IRCCS Istituto Ortopedico Rizzoli, 40136 Bologna, BO, Italy; stefano.zaffagnini@unibo.it

**Keywords:** total ankle arthroplasty, radiostereometric analysis, osteoarthritis, mobile bearing

## Abstract

Ankle osteoarthritis (OA) represents a significant social burden and is one of the main causes of chronic disability in a rapidly growing part of the world’s population. Total ankle arthroplasty (TAA) has become increasingly popular despite the poor results obtained with the first dedicated designs. The purpose of this paper was to evaluate the ankle kinematics, in vivo and under weight-bearing conditions, of a TAA through a dynamic model-based radiostereometric analysis (MB-RSA). The clinical evaluation was performed by administering the American Orthopaedic Foot and Ankle Society ankle–hindfoot score and Short Form-36 questionnaires. The kinematic evaluation was conducted through MB-RSA during the execution of an open kinetic chain and a closed kinetic chain motor task. Double radiographic images of the ankle joint were processed using dedicated software to obtain a 3D reconstruction of the ankle prosthetic components’ motion. Eighteen patients (five females) completed the clinical and instrumental preoperative and postoperative evaluations (age 59.1 ± 10.3). All clinical scores showed a marked improvement (*p* < 0.005). During the closed kinetic chain motor tasks, the ankle showed a total range of motion (ROM) in dorsi-plantarflexion of 19.84°. The parameters in varus–valgus were recorded. Physiological motion can be achieved in TAA, characterized by a wide range of motion and coupling of movements on the three planes. The results of the present work may help to understand the real movement of a widespread TAA model and possibly to improve future designs and instrumentation.

## 1. Introduction

Ankle osteoarthritis (OA) represents a significant social burden and is one of the main causes of chronic disability in a rapidly growing part of the world’s population. The degenerative articular pathology affects about 15% of the world’s population of which about 1–2% is affected by ankle OA [1,2,3,4].

Ankle arthrodesis has been and still is the gold standard for advanced stages of ankle OA, with good results in the medium-to-long term although they are not optimal [3,4,5,6,7]. In this scenario, TAA is becoming increasingly valid among surgical therapeutic options for ankle OA [8]. Newer designs have attempted to replicate normal ankle anatomy and kinematics, ligamentous stability, and mechanical alignment. The last generation designs incorporate a third component: an independent polyethylene inlay, or mobile meniscus. The “non-anatomical” design is characterized by a talar component of variable geometry and is congruent with the respective surface of the inlay, to ensure a stable coupling at this level, and a flat tibial component is present that allows the tibial surface of the insert to slide at this interface.

The understanding of the relative motion of the prosthetic components is therefore crucial to improve the sub-optimal results obtained with the first dedicated designs and assess the efficacy of the newest ones [8]. Model-based dynamic radiostereometric analysis (MB-RSA) has already been proven as a useful tool for the evaluation of total joint prosthesis kinematics in vivo and under the weight-bearing condition [9,10,11]. MB-RSA can be used to assess in vivo 3D kinematics of the prosthetic components with sub-millimetric accuracy for both rotations and translations [12]. Model-based RSA represents today a remarkable technological and diagnostic advance in this field of knowledge, allowing for high-precision and -reliability evaluations. In the literature, only a few studies have investigated the overall kinematics in vivo in the TAR.

The purpose of this study was to evaluate the overall prosthetic kinematics in vivo and under weight-bearing conditions of a TAA model through MB-RSA. The TAA model investigated was composed of three components, with a flat non-anatomical tibial surface and anterior approach, that the authors intended as a generic model representing most of the designs commercially available today. The hypothesis was that, except for surgical technical errors, the ankle arthroplasty would show intrinsic stability and allow for a range of motion associated with a physiological coupling of the six movements in the three planes of space.

## 2. Materials and Methods

Ethical approval was sought and granted by the local research Ethics Committee on April 2020 (Prot. no. 139/2020/Sper/IOR). Informed consent was obtained from all patients before the surgery was scheduled, following the principles of the Declaration of Helsinki. All study data were treated with maximum confidentiality.

### 2.1. Study Population

All patients who presented at IRCCS Istituto Ortopedico Rizzoli in Bologna, between May 2020 and September 2021, and affected by ankle OA were screened. 

The patient, according to the surgeon, decided whether conservative or surgical treatment was appropriate, as per usual clinical practice. If a surgical indication was given, they were potentially eligible to take part in the study. Overall, 34 patients affected by ankle OA and candidates for TAA were enrolled. 

The inclusion and exclusion criteria, on which recruitment took place, are summarized in Table 1. This study was performed by prospectively collecting preoperative (clinical outcomes) and postoperative (clinical and kinematic outcomes) data for all treated patients. Radiographic grading of degenerative ankle arthropathy was performed by the classification proposed by Giannini S. et al. [13].

All surgeries were performed by the same orthopedic surgeon, with extensive experience in foot and ankle surgery. The final follow-up was placed at 10 months postoperatively.

### 2.2. Surgical Instrumentation and Technique

This study examined patients who underwent ankle TAA surgery through the implant of the Exactech Vantage^®^ prosthetic model (Gainesville, FL, USA), with a mobile-bearing and anterior approach.

Skin incision is performed 1 cm lateral to the tibial crest, starting 6–8cm proximal and ending 6 cm distal to the articular line of the ankle joint.

After gentle dissection of the soft tissues, the capsule of the ankle joint is then widely opened in order to show the entire joint, from the medial to lateral malleolus. Osteophytes can be removed from the tibial and talar border of the joint using an osteotome, enhancing the exposure. Ankle alignment is determined by positioning the “alignment guide”, with an attached Tibial Cutting Block. The bone cuts on the tibial and talar side, prosthesis preparation, and positioning were performed assisted by an image intensifier. Trial components point out the correct position of the prosthesis, the appropriate size of the implant, and also the stability and range of motion (ROM). Once the position and the size of prosthetic components are defined, the final components can be implanted with a press fit fixation. As the last step, the trial mobile insert is positioned, and the proper size is chosen considering the ligamentous balance [14].

In the case of Achilles tendon retraction, lengthening to the myocardial junction was performed according to Strayer’s technique, as an accessory procedure [15].

### 2.3. Postoperative Protocol

Postoperatively, a walker boot was placed for four weeks and partial weight-bearing was allowed. During the stay, patients were instructed about the periodic removal of the walker boot to perform active and passive mobilization of the ankle. Three weeks after surgery, a progressive increase to a full load, protected by a walker boot for another week, was granted.

### 2.4. Clinical Evaluation

The clinical evaluation was performed by administering, preoperatively and postoperatively (10 months after TAA), the American Orthopaedic Foot and Ankle Society (AOFAS) ankle–hindfoot score [16,17] and Short Form-36 (SF-36) questionnaires [18,19].

### 2.5. Kinematic Assessment

**Figure 1:**
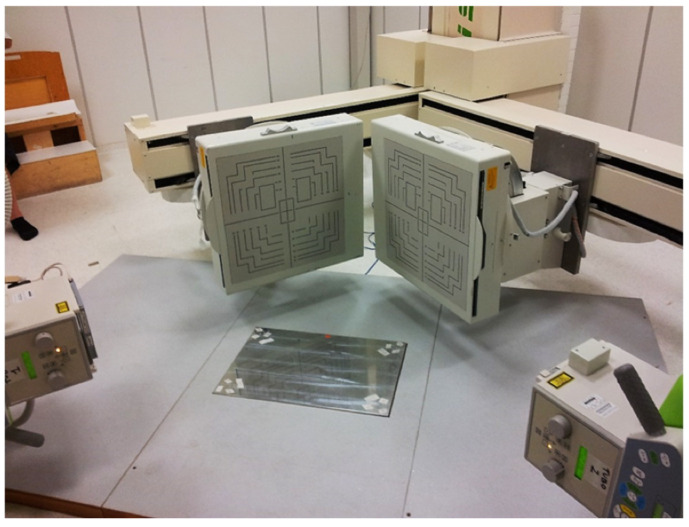
MB-RSA setting.

The MB-RSA assessment was performed through two radiographic tubes and two digital panels perpendicularly positioned that were synchronized to acquire two contemporary sets of images [12] (Figure 1). A single operator post-processed the images using MATLAB software (R2020b, MathWorks Inc., Natik, MA, USA). At this stage, the contours’ segmentation and CAD model positioning of the tibial and talus components were performed [20]. 

**Figure 2:**
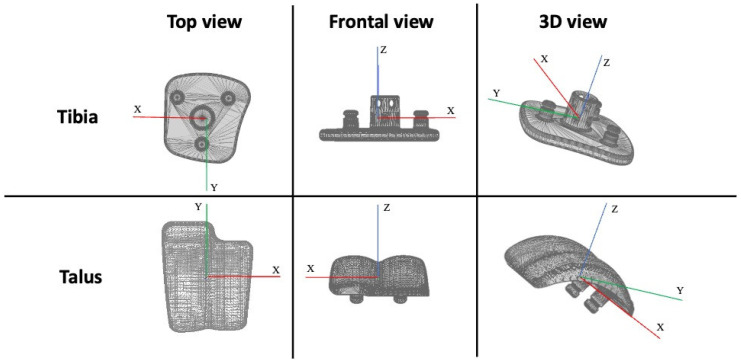
CAD reference system.

The acquisition rate was set to 8 frames per second. The movements have been described concerning the tibia in relation to the talus and polyethylene (the latter was radiotransparent and considered congruent with the talus). The reference systems are shown in Figure 2. The X axis has been used for the evaluation of the dorsi-plantarflexion angles and the medial-lateral translation, the Y axis for varo-valgus rotations and anterior-posterior translations, and the Z axis for the evaluation of internal and external rotations. The quantitative kinematic data for each patient were calculated using the Grood and Suntay decomposition [21]. According to the ISO-5725 regulation [22], the measurement accuracy of the validated MB-RSA software (MATLAB software) was 0.22 ± 0.46 mm for the model position and 0.26° ± 0.2° for the model orientation [12,20].

### 2.6. Motor Tasks’ Descriptions 

**Figure 3:**
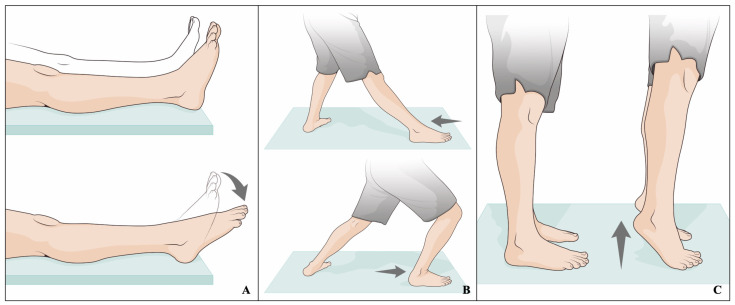
Artwork representation of the three motor tasks. (**A**) Open kinematic chain range of motion; (**B**) closed kinematic chain range of motion; (**C**) climbing on tiptoes.

As per TAR-RSA protocol [23], the motor tasks identified included “Total range of motion with open kinetic chain”, “Total range of motion with closed kinetic chain”, and “Climbing on the tiptoes” (Figure 3).

Total range of motion with open kinetic chain: The subject was sitting on a chair and was asked to perform an active mobilization of the operated ankle in dorsi-plantarflexion, without constraints or bearing weight.

Total range of motion with closed kinetic chain: The subject was standing on the ground with the non-operated ankle behind the other one. The subject started from the maximum plantarflexion position and reached the maximum dorsiflexion position with the operated ankle.

Climbing on tiptoes: Starting from the standing position, the subject was asked to climb on tiptoes.

The motor tasks evaluated during the RSA tests were identified through biomechanical objectives, clinical and surgical experience of researchers, and were based on the typical functional requirements of patients undergoing TAA. 

### 2.7. Statistical Analysis

The size of the population sample was determined by power analysis in 20 patients. All variables used for the sample description have been expressed in terms of mean, standard deviation (SD), and median. The normality of the distribution for each variable was examined in advance by applying the Shapiro and Wilk normality test. For the calculation of the difference between paired samples, the non-parametric Wilcoxon signed-rank test was used. For correlations between linear and monotone value groups (OA grade, RSA range, and clinical scores), the Pearson test and the Spearman non-parametric test were used. For all tests, a *p*-value < 0.05 was considered significant.

Statistical analysis was performed using the IBM Statistical Package for the Social Sciences (SPSS) software version 25.0 (IBM Corp. released in 2017, IBM SPSS Statistics for Windows, Vers. 25.0, IBM Corp.: Armonk, NY, USA).

## 3. Results

Among the 34 patients eligible, 14 were excluded (10 did not meet the pre-defined inclusion criteria and 4 did not consent to participate in the study). 

In total, 20 patients were selected, properly informed, and enrolled. At the time of the final follow-up (10 months postoperatively), 2 patients were excluded from the analysis (1 patient underwent a prosthetic component removal for periprosthetic infection and 1 patient requested to be excluded from the study after it had already started), with a drop-out rate (10%) lower than the average described in the literature for prospective studies [24].

Therefore, the number of patients undergoing a complete trial with clinical and instrumental assessment at the final follow-up was 18 (13 males and 5 females) with an average age of 59.1 ± 10.3 years (range 42–74 years).

Of these 18 patients, 10 were subjected to concomitant Achilles tendon lengthening according to Strayer’s technique and 14 had the picture of a clinically detectable retraction. Two patients were found to be suffering from grade 1 OA (G1), nine from grade 2 (G2), and seven patients from grade 3 (G3), according to Giannini et al. [13].

### 3.1. Clinical Evaluation

**Figure 4:**
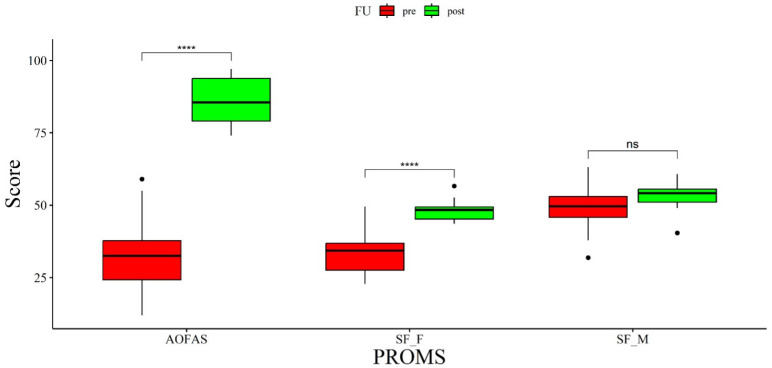
Boxplot of preoperative and postoperative clinical outcomes. ****: *p* < 0.001, ns: non-significant.

The clinical AOFAS ankle–hindfoot scores and functional (F) domain of SF-36 showed a statistically significant improvement between preoperative and postoperative assessments (*p*-value < 0.005). The AOFAS increased from a mean value of 33.7 ± 13.7 (range 12–59) to an average value of 85.9 ± 8.3 (range 74–97). Concerning the SF-36 score, the physical domain showed an improvement from preoperative scores of 33.3 ± 7.37 (range 22.8–49.5) to postoperative scores of 48.2 ± 3.4 (range 43.6–56.6), while the mental domain increased from 49.1 ± 7.5 (range 31.9–63.1) to 53.25 ± 4.53 (range 40.4–60.7) (Figure 4). AOFAS also showed a substantially overlapping improvement between patients undergoing arthroplasty and Achilles tendon lengthening (from 32.4 ± 14.7 preoperatively to 85.2 ± 7.9 postoperatively) and those undergoing TAA without associated procedures (from 35.2 ± 12.8 preoperatively to 86.8 ± 9.2 postoperatively).

### 3.2. Kinematics Description

The total ROM with a closed kinetic chain showed an average overall dorsi-plantarflexion of 19.8° ± 9.3 (range 8.1°–38.4°). If we consider the ankle neutral position as the starting point, the range of dorsiflexion was 14.6° ± 7.4, while the range of plantarflexion was 5.2° ± 5.5.

**Figure 5:**
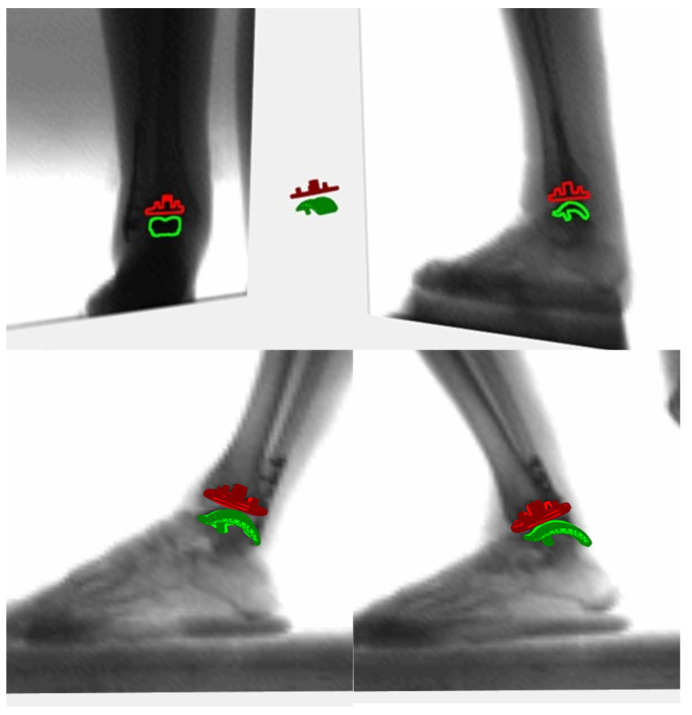
Three-dimensional reconstructions of prosthetic components in vivo and of the overall range of motion with closed kinetic chain. The graphic result was possible thanks to the use of CAD models of the prosthetic model.

Concerning the internal–external rotation, the mean value of the range was 6.3° ± 5.2 (predominantly in external rotation 5.6° ± 6.3 during dorsiflexion), while the varus–valgus range was 3.8° ± 1.6 (predominantly in valgus 2.6° ± 2.6 during dorsiflexion) (Figure 5). An average value of 3.7 mm of linear translation along the flat surface of the tibial component from anterior to posterior (starting from a position in maximum plantarflexion) was found.

The total ROM with an open kinetic chain showed a lower overall movement range than the closed kinetic chain ROM. The overall mean dorsi-plantarflexion showed a range of 20.0° ± 6.3 (range 11.7°–31.9°), specifically characterized by a dorsiflexion component of 11.1° ± 6.5 (range 2.2°–23.7°) and a plantarflexion component of 7.4° ± 7.2 (range 1.0°–18.2°). The varus–valgus and intra–extra rotation components during the overall range of motion substantially overlapped with those of the kinematic closed chain.

**Figure 6:**
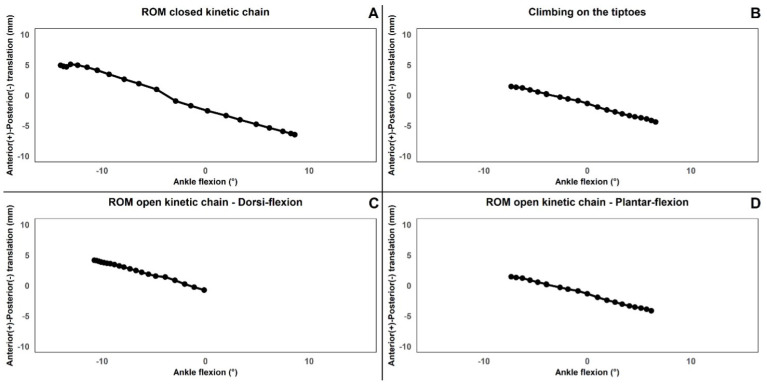
(**A**) Total range of motion with closed kinetic chain; (**B**) climbing on tiptoes; (**C**) total range of motion with open kinetic chain—dorsiflexion phase; (**D**) total range of motion with open kinetic chain. The antero-posterior translation in mm (*Y* Axis) represents the angular distance of rotation and not the anteroposterior migration of the polyethylene.

Finally, concerning the kinematics during the climbing on tiptoes (in single-stance on the operated limb), only the active closed chain movement was evaluated, showing an ankle plantarflexion average value of 14.5° ± 6.2 (range 8.34°–25.9°), superior to the that recorded during the previous motor tasks (Figure 6). This value is probably due to the forced and almost exclusive activation of the posterior flexor apparatus. The rotation and varus–valgus motion components showed an average value of 4.9° ± 2.9 and 5.1° ± 2.5. The RSA mean values are summarized in Table 2.

### 3.3. Interaction between Clinical and Kinematic Outcomes

For patients undergoing Achilles tendon elongation, a significant difference was found only in terms of preoperative SF-M, which was lower in these patients (*p* = 0.006).

The degree of osteoarthritis (OA), as a grouping variable, showed a statistically significant correlation in terms of overall open kinetic chain movement range (patients with grade 2 osteoarthritis show a higher ROM in free discharge mobilization, compared to patients with grade 1 and 3 osteoarthritis), while the average postoperative SF-36 M score correlates positively with the degree of maximum open kinetic chain plantarflexion (IC 0.469, *p* = 0.036).

The rate of improvement in postoperative clinical outcomes correlates exclusively and sensitively with the overall range of motion in RSA (plantarflex [IC 0.598, *p* = 0.009], rotations [IC 0.538, *p* = 0.021], and varus–valgus [IC 0.513, *p* = 0.03]) with closed kinetic chain.

In addition to such monotone correlations, another statistical correlation has been highlighted for the delta pre-post AOFAS and SF-36 M associated with plantarflexion in closed kinetic chain RSA (IC 0.405, *p* = 0.025 and IC 0.395, *p* = 0.028, respectively).

## 4. Discussion

TAA is gaining more and more consensus and credibility as a treatment for ankle OA. For this reason, an overall kinematic evaluation of the prosthetic components, once implanted in the patient, is of fundamental importance for the understanding of the implant characteristics. 

The objectives of this prospective study were to analyze the behavior of the individual prosthetic components in vivo and correlate this data with clinical outcomes. The initial hypothesis, that the TAA with an anterior approach, three components, and flat tibia is stable and allows for a physiological coupling of the movements on the three planes of the space, has been confirmed by the results of this study.

The results of the RSA evaluation confirmed the range of motion expected by prosthetic components in vivo. The average ROM on the sagittal plane showed values ranging from 19.9° to 14.5°, depending on the motor task performed by the patient. Among the ranges of motion on the three planes of space that have been recorded and analyzed, the most interesting and directly correlated with the gait function is certainly the dorsi-plantarflexion. In addition, the results obtained showed that movements on the six spatial components are physiologically coupled together. The low range of motion in varus and valgus denotes intrinsic stability of the prosthetic construct.

Only a few studies in the literature performed a kinematic analysis using 3D videofluoroscopy [9,25,26,27], but no purely kinematic-focused trial in vivo was performed using MB-RSA for an anterior approach, mobile bearing, flat tibia ankle arthroplasty, to the best of our current knowledge. RSA has instead been typically used, in the last two decades, for the demonstration of early migrations, for the prediction of possible mobilizations, and the analysis of micromovements at the bone–prosthesis interface of various joints but without focusing on the movement relationships between the prosthetic components [28,29,30,31,32,33,34]. Similar studies to perform an equal comparison of the data collected in this study are therefore not present in the literature.

The study that comes closest to the focus and methodology of this work was published in 2012 by Cenni et al. [9], in which the authors evaluated by 3D videofluoroscopy the range of real movement in vivo of a mobile bearing TAA model. The range of motion was slightly lower than those reported in our research, with mean values on the sagittal plane that oscillate, depending on the motor tasks required and the follow-up, from 18.7° ± 6.6 to 15.3° ± 6.9. In the closed kinetic chain movements, at a more similar follow-up to ours (12 months), an average dorsi-plantarflexion range of 16.8° ± 9.3 was reported, lower than that recorded in this study. Although the prosthetic model considered by Cenni and colleagues was also a three-component mobile bearing model, it is the first three-component TAA model designed to respect the ligamentous isometry of the ankle (BOX Ankle^®^, Finsbury Orthopaedics Limited, Leatherhead, UK) [9]. This model is characterized by a tibial component with a convex spherical surface corresponding to the proximal surface of the insert, while the talar component is represented by an anticlastic surface. The higher range of movement, as reported in this work, could be justified by the different rationale of the design itself. Where the classical prosthetic models with a mobile insert and non-anatomical flat tibia do not seem to specifically allow for the respect of the ligamentous isometry of the ankle, they are still characterized by a greater freedom of sliding of the insert on the tibial surface, since it can increase by some degree the range of movement in plantarflexion and dorsiflexion.

However, it is interesting to note the dynamic radiological evaluation of a two-component model, which also showed a lower range of motion than the values discussed above. Lenz et al. [35] analyzed the range of motion of a Zimmer Trabecular Metal^®^ Total Ankle (fixed bearing with lateral approach TAA) using biplanar videofluoroscopy, with subsequent 3D reconstruction based on a CT scan, acquired during a complete gait cycle. The range of motion recorded by the authors was 10.3° ± 3.1 on the sagittal plane, 2.5° ± 0.6 on the frontal plane, and 4.8° ± 1.7 on the axial plane.

From the kinematic point of view, the RSA angular results obtained during this research project highlight all six degrees of freedom, data consistent with the mobility of a native ankle, as recently described by Roach et al. [36], which showed considerable degrees of movement even in intra–extra rotation and inversion–subversion. Our data were quite similar, in particular with a closed kinetic chain (considered the motor task closer to normal walking), in some cases almost overlapping, to those described in the healthy ankle during the physiological gait cycle by Roach and colleagues. The latter has recorded an average dorsi-plantarflexion of 14.9°, an average overall rotation of 6.9° and a varus–valgus of 4.6°. Other authors confirm these data, describing a stance phase characterized by 15°, 8°, and 8° on the sagittal, axial, and frontal planes, respectively. However, the range of movement obtained in all these studies, including this paper, is lower than the forced passive ROM of a healthy ankle. As described in several publications, the total ROM allowed by a native articulation can reach a value between 65° and 75° on the sagittal plane and approximately 35° on the frontal plane [37,38,39,40]. The obvious difference, compared to the actual range of motion granted by an ankle arthroplasty, must not, however, reduce expectations, if between the values reported in previous studies, since physiological walking needs a degree of articulation much lower than the maximum allowed.

The ROM of patients undergoing TAA, as described in the literature, often appears much greater than the values reported in this and other similar studies by method and main topic. Ruiz R et al. described an average postoperative ROM on the sagittal plane of 33.9° ± 11.3 [39]. Other authors showed an even greater mean sagittal ROM, with values exceeding 40° [41,42,43]. The same authors of this paper, in 2021, reported mean values of the range of motion on the sagittal plane higher than 40° for the same prosthetic model used in this study [14]. These studies are characterized, like many others, by a clinical, macroscopic assessment of the ROM of the ankle, performed by goniometric measurements, with mechanized devices or dynamometers, which often make them operator-dependent and poorly defined. During the mobilization of the ankle, whether active or passive, the range of movement of the tibio-podalic complex on the sagittal axis is composed of the ROM of the ankle added to that of the adjacent joints. The component of distal movement, generated by the midtarsal joint (Chopart’s and Lisfranc’s joint complexes and navicular-cuneiform joint), can reach 20°–25° on the sagittal plane in healthy articulations, as evidenced by Takabayashi and colleagues [44]. This would confirm the hypothesis that many of the clinical measurements commonly presented among the clinical outcomes of ankle prostheses are the result of a sum of complex joint movements. In this regard, it would be useful in the future to perform MB-RSA studies that also include the segmentation of the adjacent bone structures to clarify the real percentage of the contribution of the various joints.

The clinical scores collected showed a clear statistically significant improvement in each of the three questionnaires administered. The pre–post delta was encouraging, being +52.2 for AOFAS A-H and +14.9 and +4.1 for the physical and mental domains of SF-36, respectively. These data are in line with, and in some cases higher than, those present in the literature [9,41,45,46,47]. This paper does not, however, have as its focus a quantitative evaluation of postoperative clinical outcomes, but the evaluation questionnaires have been collected considering the possible correlations between kinematic and clinical data. Interestingly, from a clinical point of view, there is a strong positive correlation between the percentage of improvement of all clinical scores and the overall range of movement with a closed kinetic chain in all three spatial planes to the MB-RSA measurements. This can be considered as a confirmation of how the kinematic characteristics of a prosthetic model can influence the patient’s perception of the replaced joint, both in purely symptomatic terms and in terms of functionality.

There are some limitations in this study. The first limitation is the reduced number of patients, included in the case series, who have completed the complete preoperative and postoperative trial. The number was also slightly lower than the power analysis conducted. Nonetheless, the vast majority of MB-RSA studies have been conducted on a sample size of 10 to 16 patients [9,48,49]. Thus, the present study represents a longitudinal assessment with one of the largest cohorts in the current scientific literature of TAA patients investigated by MB-RSA. The second limit is represented by the short-term follow-up that does not allow for the estimation of a possible tendency of improvement or worsening over time in the kinematic characteristics in patients who underwent TAA. It is important to clarify in any case how the objective of this paper was a purely kinematic assessment of the prosthetic ankle, not analyzing any early migration of the components to the bone-to-prosthesis interface, aspects that would require a longer follow-up. A third limitation can be traced back to the use of a single prosthetic model with an anterior approach that, although it represents the most widespread design, can differ in terms of movement on the three planes of space compared to other types of designs. A fourth and final limitation of this work is represented by the degree of variability of the RSA values recorded in the three planes of space. This can be traced probably to various intra-operative factors; in particular, the positioning of the components can play a role. Further future investigations are recommended, particularly using other motor tasks such as a complete step cycle or walking up and down stairs. Also, other prosthetic models can be evaluated, for example, two-component or fixed-bearing models, or additional data can be correlated based on OA grading.

## 5. Conclusions

In conclusion, this study was the first to use MB-RSA to describe the 3D in vivo kinematics and motion of a TAA model with three components and a flat tibia. At the time of the clinical study, it highlighted how this type of prosthesis design restores a satisfying ROM, associated with excellent clinical outcomes. Considering these data, the value of the MB-RSA becomes more evident. Physiological motion can be achieved in TAA, characterized by a wide range of motion and coupling of movements on three planes, following a biomechanical concept present in other models and particularly widespread in orthopedic practice. The results of the present work may help to understand the real movement of a widespread TAA model and possibly improve future designs and instrumentation.

These data can also be useful for any changes to existing designs or instruments, to increase the range of movement or to improve the positioning of the final components. The evaluation of these characteristics may also be included in a re-evaluation of surgical indications, such as patient type and OA grading. By the systematic use of this study protocol, future comparisons among different implants can be performed, thus contributing significantly to the improvement of TAA design.

## Figures and Tables

**Table 1:** Inclusion and exclusion criteria of the prospective study.

Inclusion Criteria	Exclusion Criteria
Informed consent signed Ankle osteoarthritis Primary TAR Age between 40 and 80 years BMI < 40 kg/m^2^ Patient physically and mentally inclined and able to perform postoperative rehabilitation plans and the required follow-up controls	Joint or extra-articular malalignments > 10° Neuromuscular pathologies End-stage knee or hip osteoarthritis (Kellgren–Lawrence > 3) Avascular necrosis or other ankle bone loss Local or systemic infections Alcoholism or drug abuse, psychosis or personality disorders Concurrent participation in any other experimental trial in the last 60 days prior to enrollment Documented acute or chronic disease that may affect life expectancy or make interpretation of collected data difficult Pregnancy confirmed by increased serum Beta-Hcg or ongoing breastfeeding

**Table 2:** MB-RSA kinematic data overview.

	Overall Sagittal ROM	DF	PF	Overall Axial ROM	ER	IR	Overall Frontal ROM	Varus	Valgus
Close kinetic chain	19.84° ± 9.34	14.64° ± 7.43	5.20° ± 5.5	6.25° ± 5.15	5.57° ± 6.27	0.68° ± 4.84	3.8° ± 1.64	1.65° ± 2.34	2.15° ± 2.63
Open kinetic chain	19.98° ± 6.31	11.14° ± 6.51	7.37° ± 7.15	6.47° ± 2.97	2.21° ± 5.48	4.27° ± 4.13	5.6° ± 1.7	1.23° ± 2.25	4.43° ± 2.3
Climbing on tiptoes	14.45° ± 6.15	/	14.45° ± 6.15	4.91° ± 2.89	2.79° ± 6.42	2.11° ± 5.11	5.12° ± 2.52	3.81° ± 2.62	1.3° ± 2.89

DF: dorsiflexion, PF: plantarflexion, ER: extrarotation, IR: intrarotation, ROM: range of motion.

## Data Availability

Restrictions apply to the datasets.
